# Using a Syndemics Perspective to (Re)Conceptualize Vulnerability during the COVID-19 Pandemic: A Scoping Review

**DOI:** 10.3390/tropicalmed9080189

**Published:** 2024-08-22

**Authors:** Yisel Hernandez Barrios, Dennis Perez Chacon, Yosiel Molina Gomez, Charlotte Gryseels, Kristien Verdonck, Koen Peeters Grietens, Claudia Nieto-Sanchez

**Affiliations:** 1 Institute of Tropical Medicine Pedro Kourí, Havana 11400, Cuba or yhbarrios88@gmail.com (Y.H.B.); or dennisperezchacon@gmail.com (D.P.C.); or yosiel.molina88@gmail.com (Y.M.G.); 2 Institute of Tropical Medicine—Antwerp, Nationalestraat 155, 2000 Antwerp, Belgium; cgryseels@itg.be (C.G.); tverdonck@itg.be (K.V.); kpeeters@itg.be (K.P.G.); 3 School of Tropical Medicine and Global Health, Nagasaki University, Nagasaki 852-8501, Japan

**Keywords:** syndemics, vulnerability, COVID-19, syndemic interactions, biosocial interface

## Abstract

Syndemics theory has been applied to study interactions between biomedical and social factors leading to the clustering of diseases. Because syndemics theory focuses on interactions that enhance risk, the concept of vulnerability is central to this approach. We conducted a scoping review to better understand how this theoretical framework helped to define, operationalize, and tackle issues of vulnerability during the COVID-19 pandemic. Original research, reviews, and opinion pieces elaborating on syndemics, vulnerability, and COVID-19, published between December 2019 and October 2022 and available from PubMed, were eligible. We analyzed 40 records and identified three framings of syndemics operating during this period: (1) interactions between COVID-19, diseases/health conditions, and specific social factors; (2) interactions between COVID-19 and social determinants of health; and (3) impacts of COVID-19 on specific populations. Emerging conceptualizations described vulnerability to COVID-19 as a systemic issue, explained the impact of COVID-19 control measures on increased vulnerability, and presented COVID-19 as a syndemic on its own. However, this theory’s potential for deepening our understanding of vulnerability during this pandemic was constrained by superficial explorations of the interactions between biomedical and social spheres, and insufficient theoretical and methodological support from the social sciences.

## 1. Introduction

Although interest in the syndemics perspective has grown over the last 25 years [1], this concept gained special traction during the COVID-19 pandemic. At the start of the pandemic, the term “syndemic” was used to describe the unequal distribution of COVID-19 disease outcomes in populations around the world [2,3,4], and later on, to refer to COVID-19 itself as a syndemic [5,6]. Syndemics theory has been applied since the late 1990s to study linkages between biomedical and social factors leading to disease occurrence in specific populations or geographical regions, so the extended use of the term during the pandemic did not come as a surprise. 

Syndemics theory is focused on understanding the synergistic effects of biomedical factors (bio interface) and social conditions (biosocial interface) in the co-occurrence and clustering of diseases [7]. Because of its focus on interactions that enhance risk, the concept of vulnerability has been core to the syndemics approach. The term ‘syndemic vulnerability’, specifically, defines a predisposition to the development of negative consequences resulting from feedback loops between biomedical and social factors. Therefore, understanding vulnerability under the syndemics perspective implies identifying upstream factors that generate both the environment and the circumstances that fuel synergistic effects, as well as the mechanisms that sustain said predisposition [8]. 

As social scientists and epidemiologists working in the field of public health, we are interested in understanding how the mechanisms underlying vulnerabilities in health are defined and incorporated into health policies and interventions. Adding a vulnerability angle to health research has proved to be useful to understand the unequal distribution of the risks and consequences of diseases and disasters [9]; more importantly, it has expanded the notion of individual risk factors to include historical contexts, conjunctural circumstances, lifetime trajectories, and differentiated conditions of exposure to specific pathogenic agents to interpret and explain health outcomes [10]. However, using the label ‘vulnerable’ to define specific groups and vast geographical regions can also lead to stigmatization, obscure institutional arrangements, and contextual elements with a direct impact on vulnerability conditions, and as a result, further increase pre-existing inequities [11,12]. The difficulties in operationalizing this concept have been studied in extensive reviews of recent public health literature [13,14].

Syndemics research is particular in this sense: while solidly established as a theoretical approach suited to study the biosocial nature of health risks, several scholars have criticized the way this theory is applied in research practice [1]. Critics have argued that the interactions between the biomedical and social realms are insufficiently explored, and that syndemics research tends to become ‘fuzzy’ when trying to explain how simultaneously occurring epidemics work together. Rather than describing causation—as it intends—it has been suggested that syndemics research could be better suited to explain disease distribution [15,16,17]. The tendency to reduce social realities into mere proxies with limited capacity to explain complex pathways involved in syndemics interactions has also been extensively criticized because of its limited explanatory capacity [18]. In addition, some argue that the critical nature of perspectives such as intersectionality or social justice is often absent from the epidemiological thinking informing syndemics research; as a result, power structures and political arrangements sustaining vulnerability often stay unquestioned [19,20]. Since some of these criticisms contradict core concepts in the original formulation of this approach, the debate about the concrete contributions of syndemics research to address inequities in health remains open [21,22]. Understanding that concrete ideas about how to address vulnerabilities in health emerge from the way they are conceptualized, this review was conceived to address two concerns common to these criticisms: how are syndemic interactions identified and studied? And more specifically, how are the interactions with social dynamics, i.e., the biosocial interface of syndemic relationships approached and explained?

Given the rapid expansion of syndemics research in the medical field during the COVID-19 pandemic and the potential for deepening our understanding of vulnerability in a context of acute risk, we conducted a scoping review to explore how the syndemics perspective contributed to defining, operationalizing, and addressing vulnerabilities in health. Three research questions guided this review: (1) Which social and environmental arrangements (determinants, factors, dynamics, and aspects) were described as part of the biosocial interface of syndemics involving COVID-19? (2) How was that biosocial interface conceptualized and studied (methodological approaches)? (3) What were the main contributions and limitations of the syndemics perspective to the conceptualization of vulnerability in the context of the pandemic?

## 2. Materials and Methods

We conducted a scoping review, the protocol of which was registered in October 2022 [23]. Where applicable, this review follows the format recommended by PRISMA-ScR (Supplementary File S1) [24]. Using the model of *concept synthesis* [25], we aimed to capture “concepts, viewpoints and ideas” defining vulnerability to COVID-19 under the syndemics perspective. 

Consequently, we identified eligible records under three criteria: (1) published as original research, reviews, and opinion pieces; (2) including the words syndemics, vulnerability, and COVID-19 in the title, abstract, or keywords; and (3) published between December 2019 and October 2022. 

Our primary data source was PubMed; additional records were identified through manual searches in the lists of references of the included records. No restrictions on language, studied conditions, or geographical locations were applied. 

In Boolean terms, the search string for PubMed was (“SARS-CoV-2” [MeSH Terms] OR “COVID-19” [MeSH Terms] OR “COVID” [TextWord]) AND (“vulnerabilities” [All Fields] OR “vulnerability” [All Fields] OR “vulnerable” [All Fields]) AND (“syndemic” [MeSH Terms] OR “syndemic” [All Fields] OR “syndemics” [All Fields]). 

All records were managed in Mendeley and imported into Covidence after checking for duplicates (https://www.covidence.org/ accessed on 12 October 2022). Two reviewers (YH and YM) independently screened titles, abstracts, and keywords. They discussed any disagreements and consulted a third reviewer (CN-S) when needed. Articles using the words ‘social disadvantage’, ‘disproportional or unequal impacts’, and ‘marginalized’ were retained considering their close relationship with the term ‘vulnerability’. Articles that focused purely on biomedical interactions or did not elaborate on the review topics (syndemics and vulnerability) beyond the initial mention (in titles, abstracts, and keywords), were excluded at the stage of full-text review.

A preliminary data extraction form was tested with 10 randomly selected records. The results of this pilot test were discussed with the review team, and the final version of the data extraction template was completed and applied to all records (Supplementary File S2). 

The data extraction was conducted in parallel by four members of the research team (YH, CN-S, DP, and YM) in three moments. First, each researcher extracted data from randomly assigned articles until they were all completed. Second, a different reviewer cross-checked the extracted data for accuracy and completeness. Subsequently, the reviewers collectively discussed and resolved any discrepancies, leading to the consolidation of the final set of extracted data in Covidence. This dataset was then used to address the review questions.

The data were exported to an Excel spreadsheet (Microsoft Office 2010) for qualitative thematic analyses. Records were categorized per type of publication (original research, reviews, and opinion pieces). All data items were thematically coded. Three researchers coded the data (YH, DP, and CN-S) and synthesized them in tables. Additional discussions were held with other members of the research team (KP, KV, and CG) to refine analytical categories. 

## 3. Results

### 3.1. Search and Selection Process

**Figure 1:**
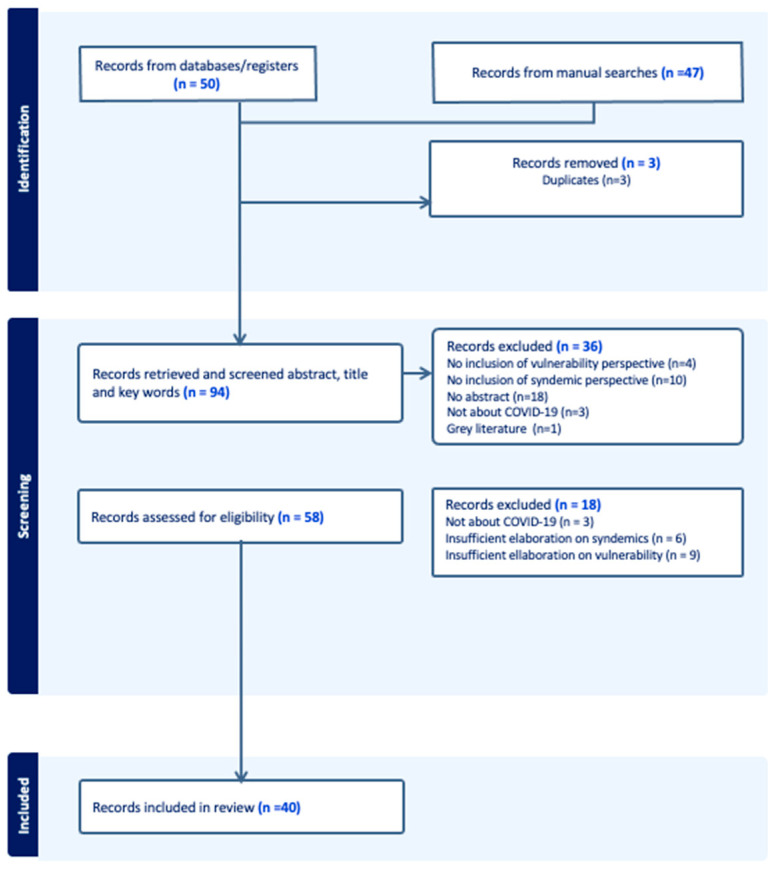
PRISMA flowchart.

Figure 1 summarizes the process of the search and selection of sources of evidence in a PRISMA flow chart. Our search strategy yielded a total of 97 articles. Three duplicates were removed in Mendeley before importing the database to Covidence. In the title, keyword, and abstract screening stage, 36 articles were deemed irrelevant. In the full-text review stage, we excluded eighteen additional articles: fifteen lacked sufficient information on syndemics and/or vulnerability, and three were not applied to COVID-19. Hence, this review includes 40 articles. 

Out of 40 articles, 13 were original research studies (conducted in the USA, Canada, England, Brazil, China, South Africa, India, and China), 17 were opinion papers, and 10 were review articles (Table 1). Of the included reviews, three were presented as narrative reviews, and one declared to follow the “guidelines of the Equator Network” [26]. The remaining records included under this category were written as essays based on extended literature reviews.

Supplementary File S3 summarizes the applications of the syndemics perspective and associated definitions or operationalizations of vulnerability identified in this review.

### 3.2. Social Factors Involved in Syndemic Interactions Including COVID-19

We identified three different framings of syndemics in this review: (a) Syndemic interactions between COVID-19 and one or several diseases or medical conditions, and specific social factors; (b) syndemic interactions between COVID-19 and the Social Determinants of Health (SDOH), and (c) syndemic impacts of COVID-19 on specific populations. In this section we summarize factors included as part of the bio–social interface within these framings.

- Syndemic interactions between COVID-19, one or several diseases or health conditions, and specific social factors

*COVID-19 and non-communicable diseases (NCDs):* Co-occurrence of NCDs and COVID-19 was the focus of seventeen records (five of them original research studies). In these cases, NCD (e.g., cardiovascular, nervous system, respiratory, kidney, and digestive diseases, as well as cancers and diabetes) were often grouped as comorbidities that, when experienced in contexts characterized by ‘socioeconomic inequalities’, ‘social vulnerability’, and ‘social disadvantage’, enhanced vulnerability to COVID-19. These contexts were generally described in terms of indicators considered to have an impact on patients’ capacity to respond to COVID-19 and its control measures. Socioeconomic inequalities, for example, were defined in terms of educational level, employment status, and income at individual, household, and area levels in India and Hong Kong [27,28]. Two publications used pre-existing vulnerability indexes to measure social disadvantage through indicators such as poverty levels, unemployment, population without health insurance, and housing crowding and ownership in specific geographical areas of the USA [29,30]. Susceptibility to COVID-19 in NCD patients was further described in terms of shared risk factors that were exacerbated during the pandemic, including sedentary behaviors and malnutrition [31,32,33,34,35]. Opinion pieces discussed changes in food intake, as well as alcohol and tobacco consumption used as coping mechanisms to deal with control measures, as concrete forms in which COVID-19 affected people living with NCD [35,36,37].

Pre-existing socio-environmental vulnerabilities were defined in terms of people’s exposure to poor sanitation systems and water and air pollutants, as well as consistently deficient access to health services. This included populations considered to be at a higher risk of NCDs, such as undocumented migrants, indigenous communities, and workers linked with unregularized activities such as illegal mining and logging [38]. Urbanization, changing lifestyle habits, climate change, and pollution were also mentioned as factors leading to adverse COVID-19 outcomes in patients with NCDs [31,33,37,39].

*COVID-19 and mental health:* The impact of COVID-19 on mental health was studied in two original research articles [40,41]. In both cases, syndemics was used as a theoretical framework to explain disproportionate mental impacts in different population groups in Canada. These publications based their results on a cross-sectional monitoring survey administered to 125,000 members of an online panel that included questions on six individual dimensions of mental health. The authors reported on the detrimental impact on mental health of COVID-19 in the general population. However, groups considered to be at higher risk of experiencing structural vulnerability due to their race, ethnicity, socioeconomic status, gender, sexual orientation, mental health, or disability reported an even higher burden of mental health issues and more difficulties coping with pandemic-related challenges. The association between COVID-19’s impacts on mental health and the pre-existing forms of social disadvantage concerning income, occupation, social support, living conditions, inequities, and emotional distress was further explored in opinion pieces [42,43,44,45]. The confluence of mental illness and substance abuse was suggested to lead to increased susceptibility to adverse consequences of COVID-19 [37,45]. Social exclusion, social isolation, and stigmatization were presented as interfering with access to health services due to the additional mental health burdens generated by the pandemic [43]. 

*COVID-19 and other infectious diseases:* Interactions between COVID-19 and other infectious diseases were also described as syndemic in nature. Specifically, interactions between COVID-19 and HIV-related comorbidities, HIV risk factors, and HIV-derived stigmatization, were considered when describing susceptibility to COVID-19 in people living with HIV (PLWH). A scoping review investigated the social and behavioral impacts of COVID-19 on this population during the first year of the pandemic [46]. The syndemics framework was used to explain the mechanisms of interaction between COVID-19 and HIV as interlocking conditions: COVID-19 psychosocial sequelae (i.e., fear and anxiety) exacerbated mental health problems and contributed to structural inequalities affecting people living with unsuppressed HIV. The co-existence of tuberculosis (TB) and COVID-19 among displaced and migrant populations was also presented as a source of dual burden. Authors suggested that pandemic control measures may have increased TB-associated risks by reducing access to health services [47].

b. Syndemic interactions between COVID-19 and social determinants of health (SDOH)

Five records described syndemic interactions involving COVID-19 and socioeconomic indicators grouped under the category of social determinants of health (SDOH). Lee and Ramírez [30] studied the associations between COVID-19 vulnerability and SDOH in Colorado. They used previously existing data on 14 social indicators including socioeconomic status, household composition, housing, and transportation in relation to health-related variables, including mental health, obesity, and substance abuse. Associations were analyzed under a syndemics perspective and the “Hazards of Place Framework” (more details on Section 3.3) to demonstrate that the overlap between mental health and chronic conditions, as well as “inequities in education, income, access to healthcare, and race/ethnicity” at the county level, exacerbated COVID-19 negative outcomes.

Similarly, Siegal et al. [48] sought to describe a syndemic between structural racism and COVID-19 by assessing disparities in selected SDOH in predominantly Black and white neighborhoods in North Carolina (USA). Differences in income levels, employment, job density, and use of public food, nutrition, and health insurance services, as well as the proximity to school-age and early childhood care, low-cost healthcare, grocery stores, and public transit were measured. The authors concluded that racially segregated communities, particularly Black communities in the USA, already experienced detrimental conditions in multiple SDOH before the pandemic and that those inequities were exacerbated by COVID-19.

Some authors proposed using a syndemics approach together with the SDOH framework to describe bidirectional relationships between susceptibility to disease and health inequities experienced by marginalized populations including the elderly, children, people with disabilities, the underinsured, the socioeconomically disadvantaged, the incarcerated, abused individuals, the mentally ill, immigrants, refugees, and racial/ethnic minorities [49,50,51].

c. Impacts of COVID-19 on specific populations

Thirteen records explored the differentiated impacts of COVID-19 on specific populations. In these cases, the authors emphasized that the conditions of marginalization, disadvantage, or exclusion experienced by some population groups, particularly racial, ethnic, and gender minorities, as well as women and immigrants—not necessarily in relation to specific health conditions—made them increasingly vulnerable to the negative health and social consequences of this pandemic. Intersections between race, gender, and occupation in the generation of marginalization were highlighted. In most cases, syndemics theory was used as a framework to explore the multiple levels of impact of COVID-19 in these populations. 

Most of the papers included in this category discussed the role of race and ethnicity in COVID-19 outcomes. Cokley et al. [52] investigated how perceptions of discrimination and police brutality influenced COVID-19 experiences for Black Americans in the USA. Perceptions of police brutality, discrimination, the COVID-19 health threat, COVID-19/race-related stress, and cultural mistrust were assessed among inhabitants of metropolitan and rural areas with high concentration of Black/African population (Black, Black American, African American, African, Afro-Caribbean, and Afro-Latinx). The authors concluded that COVID-19 concerns were further exacerbated by police violence and poor mental health, which could also have resulted in low vaccination uptake in this population. Concurrently, precarious living conditions experienced by racial and ethnic minorities due to inequities in income, working conditions, access to health care, and housing were considered to be leading to negative COVID-19 outcomes [53,54]. Disparities were described for racial minorities and immigrants employed in crucial sectors such as healthcare [55], hospitality services [45], and transportation [34]. Black women and birthing people (BWBP) and older adults experiencing pre-existing precarities and racial inequities were also presented as populations at risk of severe symptoms and worse COVID-19 outcomes due to their limited access to health care and interruption of services caused by control measures [44,56]. The pervasive effect of racism, race-associated social and health inequities, and racial injustice on health outcomes was extensively described in these publications.

The relationship between occupation, race, gender, and COVID-19 was further explored by Rogers et al. [57], who used syndemics theory as an interpretative lens to study the structural disadvantages putting street-based sex workers in New England at higher risk of COVID-19. Researchers collected data on race, ethnicity, gender, gender identity, sexual orientation, and housing status, and documented changes in sexual and food consumption behaviors during the pandemic. This study concluded that street-based sex workers were at higher risk of COVID-19 and its social impacts due to co-occurring risk factors such as homelessness, food insecurity, mental health problems, substance use disorders, and STIs/HIV. Similarly, Sönmez et al. [58] explained that immigrants represent an important proportion of the workforce of the hospitality services in the USA, and as such were severely impacted by mobility restrictions that put them out of their jobs or reduced their sources of income. The authors argued that immigrant populations linked to these economic activities experienced syndemic risks derived from socioeconomic inequities and excess chronic stress. 

Finally, gender-differentiated impacts of COVID-19 were studied by Neto et al. [59] and Duby et al. [60]. The former [59] argued that COVID-19 could have been experienced as a syndemic by gender and sexual minorities in Brazil due to various forms of vulnerability they have historically faced, including racial and gender discrimination, low education levels, precarious working conditions, and reliance on social support systems. The latter [60] took a similar approach to study the impacts of COVID-19 on adolescents, girls, and young women (AGYW) in six districts in South Africa. Thus, syndemics theory was applied to understand how pre-existing situations of poverty, unemployment, food insecurity, and domestic violence in these populations were exacerbated by the pandemic. The authors claimed that their vulnerability was not only derived from gender or age but also from ongoing mental health stressors associated with lack of social support and economic stability; therefore, they proposed to apply an intersectionality lens in combination with syndemics theory to account for the intersecting identities impacting disease outcomes in specific populations.

### 3.3. Which Methodological Approaches Were Used to Describe These Syndemics?

Out of the thirteen original research studies identified in this review, eight collected primary data [27,40,41,52,57,59,60,61], four relied exclusively on secondary data [28,29,30,38], and one combined secondary and primary data [48]. None of the identified publications intended to demonstrate the existence of a particular syndemic; instead, they used syndemic theory as an interpretative lens (an approach, a theory, a concept, or a framework) to conceptualize and analyse emerging data.

*Primary data.* Five studies reported on data exclusively collected through surveys [40,41,52,57,59], one reported on qualitative data [27], and two more conducted online or telephonic interviews in addition to surveys [60,61]. Participants were drawn from ongoing cohorts organized to follow up on the health needs of the general population [40,41] or specific groups, including Black/African adults in the USA [52] and Brazilian LGBT+ [59]. Respondents were also identified through pre-existing networks and social interventions [57,61]. In all cases, data collection focused on understanding impacts of COVID-19 on specific populations and social groups, as well as their particular needs during the pandemic. Only one group of authors reported on multiple administrations of the same survey [40,41]. Quantitative results were analyzed using binary and multivariate logistic regression models as well as descriptive statistics, while qualitative results relied mainly on thematic coding.

*Secondary data.* Four studies worked with secondary data. In all cases, researchers used publicly available data collected by health institutions to track COVID-19 incidence, prevalence, and/or mortality rates. Two groups of authors analyzed individual data on NCD pre-existences and mental health indicators to characterize dynamics at the area levels. Using data on cases and deaths from COVID-19 published by the Amazonas State Health Department, Daboin et al. [38] analyzed different municipalities of the Brazilian Amazon region and explored multifactorial correlations between sex, age, indigenous ethnicity, and COVID-19 outcomes. These factors were individually studied and then extrapolated to community (municipality) conditions in relation to poverty, sanitation, and environmental degradation to conclude that “the impact of COVID-19 in the Amazon (…) may present characteristics of a syndemic due to the interaction of COVID-19 with pre-existing illnesses, endemic diseases, and social vulnerabilities”. In Hong Kong, Chung and co-authors explored the distribution of severe COVID-19 cases in urban settings in relation to their socioeconomic position and pre-existent multimorbidities [28]. Researchers used the reported address of COVID-19 confirmed cases as published by the Centre for Health Protection (CHP) of Hong Kong to produce “area-level income-poverty rates as the proxy measures of their socioeconomic position”. 

As mentioned before, social factors were assessed using available census data or previously developed social vulnerability indexes (SVI) [29,30]. An SVI developed by the US Centers for Disease Control and Prevention (CDC) to identify counties particularly vulnerable to environmental disasters in 2018 was used in two publications. In order to characterize health and social vulnerabilities in Colorado, Lee and Ramírez [30] used three different indexes: the CDC’s SVI to track specific social determinants (economic stability, education, community, and social context); the health vulnerability index to track underlying health conditions such as diabetes, obesity, and mental health at the county level; and a third index developed during the study to show interactions between these two domains and COVID-19 burden rates. Islam et al. [29] also used the SVI to determine social disadvantage at the county level; these data were included in a model of joint distribution of COVID-19 mortality and five chronic conditions (obesity, diabetes mellitus, chronic obstructive pulmonary disease, heart disease, and chronic kidney disease) in the most vulnerable counties. Neto et al. [59] adapted a vulnerability index previously applied to the LGBT+ population to assess personal and social vulnerability to COVID-19. This index measured what they defined as three vulnerability dimensions: income (defined as living with minimum salary or no income before the pandemic); COVID-19 exposure (described in relation to adherence to preventive measures and contact with people diagnosed with COVID-19), and health (including indicators such as being a user of the public health system and being previously diagnosed with an NCD). 

Geo-referenced data were also used to explore clustering of COVID-19. In the USA, Siegal et al. [48] used GIS data (compiled in open mapping and transportation databases) to measure and compare distance to public facilities before COVID-19 in racially segregated communities. The authors proposed a place-based methodological framework to generate “contextually-informed, data-driven and cross-sector responses”. Similarly, Lee and Ramírez [30] used data available at the county level in Colorado to explore associations between SDOH and COVID-19 incidence. Due to the absence of data on COVID-19 distribution, the authors used a spatial interpolation model (Empirical Bayesian Kriging) to estimate census tract-level rates of COVID-19. A Hazards of Place Framework was used to identify clusters or “hotspots of persistent risk” in mountainous and urban areas of central and southern counties in the state. 

### 3.4. Conceptualizations of Vulnerability under a Syndemics Perspective

Different definitions of vulnerability around syndemics were identified in the literature reviewed (Supplementary File S3). Most definitions focused on explaining higher risk of infection, illness, or death by COVID-19. In this section, we present three aspects of the vulnerability concept that were particularly salient in the literature about COVID-19 and syndemics: (a) descriptions of vulnerability as a systemic issue, i.e., implying multiple levels and types of interactions; (b) the role of COVID-19 control measures in the generation of new forms of vulnerability; and (c) conceptualizations of COVID-19 as a syndemic in itself. We conclude this section with a summary of theoretical and programmatic discussions proposed around issues of vulnerability in different publications.

(a) Vulnerability as a systemic problem

Multiple authors advocated for the use of the syndemics perspective as an application of systems’ thinking when researching vulnerabilities associated with COVID-19. These systemic views were interpreted as wider definitions of health [33] in which disease occurrence cannot be dissociated from the specific context in which it emerges [31]. The syndemics framework was used to describe a relationship in which COVID-19 ‘increased’, ‘visualized’, ‘hindered’, ‘deepened’, ‘exacerbated’, ‘reinforced’, or ‘perpetuated’ pre-existing conditions of social disadvantage, or ‘generated’ emerging vulnerabilities around the public health measures implemented to control the pandemic. While most articles described bidirectional (mutually reinforcing) relationships between biomedical and social factors leading to syndemic outcomes or occurring in syndemic contexts, two groups of authors engaged in discussions about the causal link between syndemic interactions and COVID-19 outcomes. Daboin et al. [38] referred to a “syndemic context” (described as the product of interactions between pre-existing diseases and social vulnerability) creating difficulties in diagnosing and treating COVID-19 in the Brazilian Amazon, and suggested a direct causal link between this context and high COVID-19 incidence and mortality in the region. Similarly, Mezzina et al. [43] discussed the multifactorial complex nature of causality in health and proposed a descriptive model in which different domains (social determinants, social vulnerability, and social inequalities, among others) act as a “web of determinants” with “non-linear and complex effects” over COVID-19 outcomes. 

Individual vulnerability was described in relation to pre-existing health and social conditions enhancing COVID-19 susceptibility. It was often mentioned that these individual vulnerabilities intersect and reinforce each other [40,60,62]. As an example, the vulnerability of girls and women was described as a result of intersecting identities that interact with their socioeconomic status (e.g., living in poverty [44]), living circumstances (in humanitarian settings [42]), income-generation activities (e.g., sex workers [57]), and health-related issues (e.g., HIV-related stigma, birthing conditions [44], mental stressors [60]) to create differentiated layers of risk in this population. While, biologically speaking, COVID-19 did not seem to particularly affect women, records included in this review described “gendered modes of transmission” derived from limited access to health care and social services, the militarization of movement, extended impacts of gender-based violence, and drastic reductions in economic resources.

Subsequently, social vulnerability was characterized as “political decisions and cultural barriers” [59] affecting the course of the pandemic. In this case, the focus of the analysis was not on the individual but on context-specific social circumstances impacting exposure to infections and the development of negative outcomes. Some authors described, for example, how the vulnerability of immigrants, refugees, racial/ethnic minorities, and indigenous communities stemmed from a lack of access to quality healthcare systems. This absence not only predisposed them to particularly negative disease outcomes but also subjected them to substandard services during the pandemic [38,50,55,63]. Thinking of health systems beyond the criteria of clinical cost-efficiency to enable special protection to particularly at-risk populations was recommended as a way to break discriminatory and marginalizing healthcare practices [53,55,56].

The living conditions under which marginalized populations concentrate were also explored in relation to socio-environmental vulnerability [28,29,33,34]. Government-controlled confined spaces such as prisons and detention centers [45,51] were considered particularly conducive to increased SARS-CoV-2 transmission and worsened health outcomes, while humanitarian settings [42,62] were described as contexts where poverty, conflict, displacement, and lack of infrastructure coincided with and co-produced particularly devastating impacts of COVID-19.

At a higher level, structural vulnerability was treated as the “locus of danger, damage, and suffering” [63] experienced by population groups according to their position within specific structures of power. This position was deemed critically relevant to understanding the impacts of COVID-19. Power structures conferring and sustaining privilege during the pandemic based on race [48,52], age [56], socioeconomic position [39], productive sector [36,45,62], and health conditions [40,41,51,63] were extensively described. The structural role of racism, as a socio-political force underlying the conditions of marginalization experienced by racial and ethnic minorities, was mentioned in all publications dealing with this topic [45,55]. In addition, several authors described the important influence of political tensions around pandemic management in countries such as the USA, Mexico, Brazil, India, and Pakistan on negative COVID-19 outcomes and the accentuation of pre-existing structural vulnerabilities [32,38,44,51,55,63,64].

COVID-19 was also analyzed as a global problem that, much like food insecurity, natural and technological disasters, climate change, and population mobility, provided evidence of emerging vulnerabilities. Although these risks were described as latent in the general population, they have also introduced particular forms of vulnerability in groups that have not been traditionally considered at risk. This included people suffering the consequences of the growing incidence of NCDs in high income countries [32,65], those whose income level relies on large-scale food chains [35], and migrants for whom legal irregularity and uncertainties about the future are conducive to increased precariousness in several areas of life, including health care and social support systems [47,51,58].

(b) Emerging vulnerabilities: COVID-19 control measures

The generalized implementation of interventions to control COVID-19 transmission, particularly social (physical) distancing, lockdowns, and mobility restrictions, was described as a COVID-19-specific form of vulnerability [3,5,21,34,40,41,44,49,50]. Among its negative outcomes, researchers mentioned (i) increased poverty and unemployment; (ii) interruption of food supply chains, particularly those involving animal-based products [31,33]; (iii) severe impacts on the population’s mental health [40,41]; (iv) increased adoption of unhealthy lifestyles and substance abuse [33,46]; and (v) alterations in the seasonal patterns of respiratory infections [31]. 

Both stay-home orders and the highly controlled movement of the population were connected to increasing gender-based and other forms of violence [45,52]. Gender-based violence (GBV) was a distinct phenomenon associated with previous outbreaks of infectious diseases such as Zika and Ebola in humanitarian settings [42,62]. It was presented as a pre-existing epidemic that often goes under the radar and worsened with the interruption of attention services due to control measures. Transferring lessons learned from previous epidemics and designing policy responses to tackle the syndemic relationships between infectious diseases and GBV was recommended.

These specific forms of damage derived from government and health systems’ responses to the emergency were considered a form of programmatic vulnerability [59]. Importantly, authors who engaged in discussions on this topic mentioned the importance of using a syndemic approach to identify populations and regions that should be prioritized in the response to pandemic threats [29,31,37,40,45,51,56,60,62,63].

(c) “COVID-19 as a syndemic” or “the syndemic nature of the pandemic”

An important feature of the scientific literature produced around the pandemic was the rapid spread of the expression “the COVID-19 syndemic”. In our review, five opinion pieces and four reviews (22.5%) adopted this term [32,33,35,37,43,49,53,64,66]. Different from previous uses of the term to describe relationships between specific biomedical and social factors, in this case, the syndemic concept was used to emphasize (a) the large scale and diversity of COVID-19 impacts on vulnerable or marginalized populations; (b) the diverse nature of factors and interactions involved in its occurrence; and (c) the idea that the pandemic was simultaneously cause and consequence of pre-existing vulnerabilities [31,32,33,35,43,44,50,66]. This framing resembles the use of the expression “the syndemic nature of the pandemic”, through which authors emphasized how multiple and extensive interactions between pre-existing social and biomedical conditions resulted in the emergence of COVID-19 [28,31,63,64]. Singer and Rylko-Bauer further explained the use of this expression by describing how COVID-19 made clear the biosocial nature of health by bringing attention to three aspects previously enounced in syndemics theory: (a) interactions between diseases and health conditions that increase overall burden at multiple scales; (b) interspecies interactions; and (c) interactions with social dynamics underlying the clustering of diseases and risks [63].

Concurrently, the COVID-19 pandemic seemed to provide fertile ground to further apply syndemics-related terminology. For example, the term ‘syndemics framework’ was often employed to explain theoretical, methodological, and analytical decisions supporting the definition of specific interactions. ‘Syndemic contexts’ were mentioned to describe the resulting composition of social and geographic circumstances in which health conditions overlap [38,59,64]. Some authors described the ‘syndemic effects’ [31,41,52,61] or the ‘syndemic outcomes’ of COVID-19 [37,50]. Finally, the term ‘syndemic vulnerability’ was referred to describe the multiple levels and nature of impacts of the pandemic on vulnerable populations [41,47,51].

(d) Theoretical, methodological, and policy recommendations on syndemics and vulnerability

In terms of theory, four reviews focused specifically on the implications of using syndemics theory in combination with other theoretical frameworks. Singer and Rylko-Bauer [63] used the theoretical lens of syndemics and structural violence to analyze how different socio-environmental configurations engendered different syndemic interactions during the pandemic, exposed the global rise of NCDs and their potential interactions with infectious diseases, and shed light over profound problems in global health systems. According to the authors, while syndemics emphasize the synergistic interactions between biomedical conditions and socio-environmental factors, the concept of structural violence adds a focus on the effects of the ‘structures of inequality’ that sustain poverty and multi-dimensional discrimination. In their words, “‘structural violence’ drives syndemics”. These authors proposed that by using these two theoretical frameworks together, both originated in the critical anthropology field, practitioners can bring discipline-specific knowledge to inform contextualized public health responses to this global crisis. 

Conceptual arguments around the idea of ‘context’ in syndemics research were explored by Pirrone et al. [65]. This group of authors conducted a literature review and expert interviews to explore how context has been defined and studied in syndemics research. Focused on syndemics involving NCD and mental health, they concluded that most studies centered on factors that affected populations at micro levels. They argued that, as a consequence, research tends to overlook structural factors shaping said contexts and, in turn, limit the potential contributions of syndemics to COVID-19 management. Since this trend is closely influenced by the methodological designs previously used in the study of syndemics, this review recommended developing longitudinal and population-level analyses that incorporate multiple disciplinary views to study the impacts of context in COVID-19 outcomes. Expanding syndemics-informed research with multi-level and transdisciplinary research designs allowing integration of different datasets was recommended by multiple authors in this review [34,44,48]. 

Concurrently, Fronteira et al. [31] described COVID-19 as “One Health issue of syndemic nature”. These authors referred to the important impacts of COVID-19 control measures on food systems, particularly in areas in which animals play a central role as food sources, income, transportation, fuel, and clothing, among others. Under this rationale, researchers advocated for “syndemic policies” to tackle interconnections between humans, animals, social, and abiotic environments engaged in COVID-19 transmission, which implies (i) learning from documented experiences; (ii) using theoretical frameworks that properly approach the multi-level, interacting, and dynamic nature of the pandemic; and (iii) identifying community responses to COVID-19.

Transfer of knowledge and integration with previous experiences of infectious diseases management was another way of bringing the syndemics angle into programmatic actions. Garcia [50] advocated for using syndemics theory in association with the SDOH framework as an opportunity to generate collaborations between social workers and the public health sector in the development of “biological-social interventions” for vulnerable populations. Additional policy recommendations identified in this review included integrating the management of COVID-19 and other respiratory diseases in migrant populations [46]; informing policies for improving working conditions and workplace regulations considering diseases with syndemic potential [57]; designing pandemic management and preparedness strategies with a focus on vulnerable and at-risk populations [41,53,63]; and formulating public health actions that are grounded in mental health promotion under an equity-oriented lens [58].

## 4. Discussion

Under the syndemics perspective, vulnerability during the COVID-19 pandemic was extensively described in relation to (a) the risk of being infected, developing illness, or dying from COVID-19, and (b) experiencing negative health, economic, or social outcomes as a result of COVID-19 control measures. Exploring heterogeneous health outcomes, particularly clustering of cases or deaths in specific populations or geographical areas, emerged as the most often claimed argument to apply a syndemics framework during the COVID-19 pandemic. 

From these results, two important contributions of syndemic thinking on vulnerability that emerged during the COVID-19 pandemic can be highlighted:

*(a) Advances on a biosocial conception of health:* All the publications included in this review elaborated on intersections between biomedical and social factors that occurred around COVID-19. A typical application of the syndemics framework during the COVID-19 pandemic would include biomedical factors considered to increase susceptibility to COVID-19, and contextualize, locate, or explain them in the light of socio-environmental factors or dynamics enhancing their negative outcomes. Another common application would describe syndemic interactions or syndemic effects of the pandemic in population groups historically exposed to marginalization based on race, ethnicity, gender, and/or migratory status. The fact that syndemics research has contributed to the definition and understanding of a biosocial conceptualization of health is an important contribution. For too long the public health community has been focusing on downstream “risk factors” and many opportunities for sustainable disease control have been missed. In this review, the syndemics framework helped to illustrate how vulnerabilities to COVID-19 overlapped with vulnerabilities to standardized COVID-19 control measures across contexts, which could provide important arguments to reflect on the profound implications of thinking of these measures simply as ‘non-pharmaceutical’ interventions. COVID-19 control measures reinforced and engendered new vulnerabilities and provided a clear example of the social ramifications of these measures beyond health-specific spheres. 

*(b) Syndemics as a language of complexity:* The fact that none of the original research studies included in this review focused on demonstrating the existence of a particular syndemic, but rather on applying this concept as a theoretical framework to analyze different datasets, might be an indication of a new phase in syndemics research. Instead of looking into the empirical validity of syndemics’s theoretical claims, researchers have appropriated its language to describe complex, i.e., multifactorial, multiscale, intricate, and multidirectional pathways involved in the generation of health and disease, and very particularly, vulnerability to disease. This coincides with the framing of COVID-19 as a syndemic. Although it has already been argued that extending the use of the term ‘syndemic’ to all the effects of the pandemic can actually limit the extent to which this perspective can identify interactions that matter amidst a global emergency [21,67], the popularity of Horton’s piece [5] (cited over 700 times since its publication) is noteworthy. Uses of this expression in this review referred to COVID-19 as cause and consequence of profound interconnections between different levels of vulnerability and, as a result, facilitated engagement with systemic views in which individual vulnerability was always embedded in larger social, environmental, and political contexts. These discussions linked COVID-19 with new forms of vulnerability at a planetary scale, in which interactions with environmental conditions, non-human species, and economic systems play a fundamental role. Some of the theoretical and policy recommendations already point to the need for interdisciplinary and multisectoral action to tackle the most pressing challenges ahead of us. Syndemics research can definitely contribute to such explorations.

However, we acknowledge that some of the criticism expressed around syndemics remained true for the publications included in this review. We want to focus here on two concrete limitations identified in this study:

*(a) Limited exploration of interactions between biomedical and social spheres:* Records frequently mentioned that COVID-19 generated multiple forms of interactions with pre-existing medical or social conditions; nevertheless, the most frequently described form of interaction was one in which COVID-19 created additional burdens in patients with comorbidities and populations experiencing different forms of social disadvantage. Although important, it could be argued that this was a somehow expected outcome of the pandemic and that other forms of interactions remained largely unexplored. For example, which interactions moderated the variability of outcomes within these already ‘vulnerable’ populations? How to account for context-specific circumstances and social responses in populations historically subjected to marginalization or discrimination? In which ways was COVID-19 different from other forms of risk experienced by these populations? Concurrently, social vulnerability or social disadvantages were often described as the main characteristics of syndemic contexts. However, is being part of the context sufficient to establish mutually reinforcing relationships between these forms of marginalization and COVID-19 outcomes? More importantly, what are the implications of considering these distinct phenomena (social vulnerability or social disadvantage) as ‘context’? 

Limited research on the types of syndemic interactions can be a result of the difficulties in conducting scientific research during the pandemic. However, it could also be argued that there are conceptual limitations in the analytical methods of mainstream epidemiology that limit the possibility of integrating non-epidemiological thinking to explain complex phenomena. As an example, multiple publications emphasized the role of place in the generation of vulnerability. In these cases, most studies focused on understanding COVID-19 clustering around pre-established administrative units (counties, zip code areas, etc.). Using geo-located data, accessed through publicly available datasets, syndemic interactions were often described in relation to the co-existence of specific comorbidities and social conditions within the same geographical space. However, is this co-existence enough to explain the role of ‘place’ in syndemic interactions? Although somehow mentioned, complex interactions between natural, built, and social environments across geographical scales remained largely unexplored according to this review. 

*(b) Limited theoretical and methodological developments from the social sciences and other disciplinary fields:* In general, socio-environmental factors were treated as ‘social’ risk factors [1,68], i.e., explored to the extent to which they increased the burden of infection and disease. Issues such as social vulnerability and disadvantage were measured through socioeconomic indicators, inherently limited to portray the material, political, and emotional impacts of complex social phenomena. This was particularly true for studies using the framing of the SDOH. When the SDOH, as a group, were included in syndemic interactions, they acted as a generic framework to account for issues of a very diverse nature, from precarity in housing to lack of political representation. They were approached by means of indicators from which we can advance, and perhaps confirm, associations between social factors and COVID-19 outcomes; however, they cannot explain the processes of social determination of health that led them to be relevant in specific contexts [69]. Consequently, and contrary to what could be expected, using the SDOH framework, as well as terms such as racism, gender-based violence, and stigmatization, without acknowledging their theoretical foundations, can obscure rather than disentangle the specific mechanisms under which they influence health and disease. While bringing the syndemics perspective is an important step towards more holistic approaches to health, it is not enough to explain the theoretical dimensions of phenomena that are social in essence and for which validated theories, methods, and knowledge exist. 

A recent scoping review by Bulled and Singer also focused on applications of syndemics thinking during the COVID-19 pandemic [70], concurring on the conceptual and methodological limitations of current syndemics research. Importantly, the authors made a strong claim to reconsider formulations that conceptualize syndemic interactions as “universal”, as the syndemics perspective was conceived precisely to explain the opposite: that health vulnerabilities are highly contextual [67]. Bulled and Singer also identified what they referred to as “misuses” of the term syndemics, when it is applied to research that characterizes independent risk factors without addressing specific interactions. Our review makes an important contribution in this regard: when researchers use the syndemics perspective to explain vulnerability, a more nuanced picture emerges. Although mostly anchored in mainstream statistical methods, all the publications included in our review build on a biosocial conception of health, which could indicate that syndemics is, indeed, a suitable theoretical device to study complex interactions. Although both reviews draw on largely different sets of records (only 14 out of 40 records included here were also included in the review by Bulled and Singer), they arrive at relatively similar conclusions. Nevertheless, we want to highlight two important differences in our findings. First, the fact that spatial analyses were more frequently used in syndemics research during the COVID-19 pandemic is not necessarily an indication of a more rigorous application of the concept. We have already pointed out the lack of engagement with spatial realities identified, which can lead to establishing associations that disregard internal social and geographical heterogeneity in large spatial units of analysis. Second, we agree with the idea that there is a conceptual movement in syndemics research, but for us, this conceptual movement builds on syndemics as a language of complexity. Our review provides a snapshot of how researchers have appropriated the term syndemics—with no evaluative interests involved. The epistemological implications of this movement constitute an interesting area for future research.

## 5. Conclusions

The syndemic perspective made multiple contributions to understanding vulnerability during the COVID-19 pandemic; nevertheless, current applications of the theory may threaten its empirical foundations and hinder the effective use of this concept in public health policies and practices. During the most recent pandemic, syndemics research advanced a biosocial conception of health that emphasized the role of socio-environmental factors in disease clustering and interaction. Syndemics served as a language of complexity, enabling researchers to describe the multifactorial and multidirectional pathways involved in vulnerability to disease. Despite these contributions, this review identified limitations in its current application. Specifically, there has been limited exploration of the interactions between the biomedical and social spheres. Furthermore, there has been a lack of theoretical and methodological contributions from the social sciences, as we observe socio-environmental phenomena primarily being treated as ‘social’ risk factors and approached through limited quantitative indicators. Potential ways of overcoming these limitations include enhancing interdisciplinary collaborations to address the complex and multifaceted nature of vulnerability during crises of global scale such as COVID-19; enriching syndemics research with theoretical and methodological developments from the social sciences to deepen the understanding of the social mechanisms through which they shape vulnerability; investigating complex associations between biomedical and social realities; and exploring methodological approaches that facilitate investigation of the intersections between natural, built, and social environments in the generation of disease. 

## 6. Limitations and Strengths of the Review 

An important limitation of this study is that we focused our search on publications registered in PubMed. We are aware that other databases—more specialized in multidisciplinary research, for example—could have yielded different results. However, we were interested in understanding the potential contributions of these concepts to the management of the pandemic in the medical field, and for that reason, we considered that PubMed was the best source. As previously mentioned, another review with a similar focus consulted a larger set of sources; our findings provide a focalized analysis zooming into discussions on vulnerability in syndemics research [70]. We also acknowledge that important conceptual discussions around the term ‘vulnerability’ have occurred in different fields, which, in some cases, have led to a reduced or limited use of the term. That is the case for HIV control and prevention, for example. This conceptual discussion could explain the limited number of HIV-related records identified in this review, despite the extended use of the syndemics perspective in this field. This could also have been the case for other expressions associated with the term vulnerability that could have escaped our search strategy. Another limitation of this study is that it included many opinion pieces, which limits our claims about the theoretical and methodological limitations of syndemics thinking in the pandemic context. 

## Figures and Tables

**Table 1:** Characteristics of the articles included in the review (n = 40).

Record Characteristics	Records	%
Type of publication		
Opinion pieces	17	42%
Original research	13	32%
Reviews	10	25%
Year of publication		
2020	6	15%
2021	19	47%
2022	15	37%
Setting/Location of study		
Global	13	32%
Specific country	25	62%
Several countries	2	5%

## Data Availability

The original contributions presented in the study are included in the article; further inquiries can be directed to the corresponding author.

## Supplementary Materials

The following supporting information can be downloaded at https://www.mdpi.com/article/10.3390/tropicalmed9080189/s1, Supplementary File S1: PRISMA-ScR checklist; Supplementary File S2: Data extraction form; Supplementary File S3: Table synthesis of results.
