# Supplementary material for: Using a Syndemics Perspective to (Re)Conceptualize Vulnerability during the COVID-19 Pandemic: A Scoping Review
Source: Trop Med Infect Dis. 2024 Aug 22;9(8):189. doi: 10.3390/tropicalmed9080189 (PMC11360217; doi:10.3390/tropicalmed9080189)
Supplement: Supplementary file 1 [file tropicalmed-09-00189-s001.zip › Supplementary file S-2_Data extraction form.pdf]

Supplementary Material S2. Final version of the data extraction form.

Heading	Subheading
General information	Journal
	Year of publication<br>o 2019<br>o 2020<br>o 2021<br>o 2022
	Type of publication<br>o Review<br>o Original research<br>o Opinion pieces
	First author
	Affiliation(s)
Setting and study characteristics	Study aim(s)
	Location
	Data collection methods
Syndemic perspective	Syndemics being described
	Type of interactions described<br>o Bio-Bio<br>o Bio-Social<br>o Bio-Socio-Environmental
Bio-Social interactions	Conceptualization (e.g., theoretical foundations, factors, indicators)
	Methods (e.g., design, disciplines involved, types of data collected)
Bio-Socio-Environmental interactions	Conceptualization (e.g., theoretical foundations, factors, indicators)
	Methods (e.g., design, disciplines involved, types of data collected)
Vulnerability perspective	Conceptualization (e.g., ceoncepts, groups, factors)
Recommendation	Research, policy and practice recommendations
Comments	Additional information to be considered by the reviewers.
