# Supplementary material for: Using a Syndemics Perspective to (Re)Conceptualize Vulnerability during the COVID-19 Pandemic: A Scoping Review
Source: Trop Med Infect Dis. 2024 Aug 22;9(8):189. doi: 10.3390/tropicalmed9080189 (PMC11360217; doi:10.3390/tropicalmed9080189)
Supplement: Supplementary file 1 [file tropicalmed-09-00189-s001.zip › Supplementary material S-3_Synthesis of results_V03_SENT.pdf]

Table S2. Main references related to vulnerability definitions, operationalization, and strategies around syndemic during the COVID-19 pandemic in the literature reviewed

No.	Record	Location	**Syndemics** – main uses of this perspective	**Vulnerability –** definitions, operationalization, and strategies
1	Chung et al., 2021 [28]*	Hong Kong	"…COVID-19 severity and related mortality arise as a result of a syndemic of COVID-19, socioeconomic, inequalities in chronic diseases, and the SDOH." Provides "an empirical investigation into the syndemic nature of COVID-19 based on the context of Hong Kong."	"Socioeconomic disadvantage, and the corresponding exposure to health-compromising SDoH, as well as the devastating epidemic and socioeconomic patterning of chronic diseases, may interact with each other to exacerbate the severity of COVID-19." "…a better COVID-19 containment and relatively equitable inpatient care could provide a safety net to the socioeconomically disadvantaged and vulnerable groups against emergency and severe COVID-19."
2	Cokley et al., 2022 [52]	USA	"Apply a syndemic approach to examine the association of general and race-related concerns about COVID-19 and police brutality with the mental health of Black Americans; the mediating role of perceived discrimination in the associations between COVID-related concerns and mental health; and the association between cultural mistrust and the vaccination decisions of Black Americans." "This approach suggests that Black people may be dealing not only with the specific event when a negative incident occurs but also their disproportionate danger due to environmental factors".	"The intersection of these pandemics has unveiled to the public(…) how pre-existing conditions in Black communities can increase Blacks' disease burden in times of public health emergencies. " "Black Americans are more likely to have pre-existing conditions that make them more vulnerable to COVID-19 infection." "Effective practices will improve not only Black mental health but also the concerns of Black people about seeking mental health services as well as their concerns about increased vulnerability when public health crises occur."
3	Daboin et al., 2022 [38]	Brazil (Amazon region)	"The impact of COVID-19 in the Amazon is not limited to the direct effects of the pandemic itself; it may present characteristics of a syndemic due to the interaction of COVID-19 with pre-existing illnesses, endemic diseases, and social vulnerabilities." "The syndemic context makes the detection and treatment of COVID-19 complex in the region."	"Existing socioeconomic discrepancies impacted the course of the pandemic, determining poorer outcomes in municipalities with highly vulnerable populations, mainly in rural areas." "…highlight the importance of systematic and persistent efforts to research and develop interventions seeking environmentally sustainable development of the Brazilian Amazon and improvements in healthcare quality and access to socioeconomically vulnerable populations."
4	Duby et al. 2022 [60]	South Africa	The COVID-19 pandemic interacted with pre-existing mental health stressors in South African adolescent girls and young women. "Syndemic theory provides a framework for understanding how overlapping and co-occurring risk factors situated in a specific social context cluster to create intersecting epidemics which combine to enhance vulnerability."	"…the COVID-19 pandemic is affecting vulnerable groups disproportionally, further exacerbating pre-existing inequalities." "Intersectional vulnerabilities, risks, and disadvantages based on characteristics such as gender, age and SES status – in the case of AGYW in this study – are produced and continually reproduced in a dynamic relationship, with a cumulative effect that exceeds the sum of the detrimental effects derived from each single characteristic."

5	Islam et al. 2021 [29]	USA	"…socially disadvantaged populations might be affected by a confluence of epidemics from chronic diseases and COVID-19, which may exacerbate each other: a concept known as 'syndemics'"	"The Social Vulnerability Index (SVI) was developed to identify counties that were most vulnerable to the social and economic impacts of major environmental disasters and as such most likely to require public assistance after such an event."<br>"The findings inform the understanding of the social determinants of COVID-19 and the utility of measures such as SVI in the public health response to the pandemic."
6	Jenkins et al. 2021 [40]	Canada	"In the context of this study, syndemics theory helps to explain why the mental health consequences of COVID-19 are more concentrated among structurally vulnerable groups, due to interactions between the virus and co-morbidities health conditions, racism, poverty, social exclusion, and discrimination."<br>"…the pandemic response would benefit by approaching COVID-19 using syndemics theory."	"Those who experience health, social, and/or structural vulnerabilities are more likely to endorse mental health deterioration, challenging emotions, and difficulties coping".<br>"Emphasis was placed on facilitating the identification of the disproportionate impacts of the pandemic on populations or groups identified as experiencing increased risks due to structural vulnerability and pre-existing health and social inequities. This was achieved by including items on race/ ethnicity, socioeconomic status, gender, sexual orientation, mental health and disability status."
7	Jenkins et al. 2022 [41]	Canada	"This study draws on syndemics theory and prior evidence regarding structural vulnerability and health inequities."<br>"Utilize syndemics theory as a framework for understanding how mental health disparities among structurally vulnerable groups are created and perpetuated by the COVID-19 pandemic."	This paper analyses "the mental health impacts of the COVID-19 pandemic, focusing particular attention on how syndemic structural vulnerabilities intersect to shape mental health inequities."<br>People experiencing pre-existing mental health conditions, disability, LGBTQ2+ identity, and being Indigenous are more likely to report mental health consequences.<br>"Results illustrate significant inequities in mental health impacts during the COVID-19 pandemic, demonstrating the potential syndemic effects of the pandemic for some structurally vulnerable populations."
8	Lee and Ramirez 2022 [30]	USA	COVID-19 vulnerability and social determinants of health as a syndemic. "In addition to social vulnerability, COVID-19 highlights the overlap of health risks in communities, that may increase vulnerability to infection and severe outcomes."<br>"This layer of vulnerability context reflects a broader public health problem amidst the COVID-19 pandemic, which some have described as a syndemic. A syndemic, in contrast to an epidemic focused on one disease, describes a public health phenomenon where multiple health and disease conditions cluster in a population alongside, and sustained by, a set of adverse social, economic, and structural conditions."	The study applied three types of indexes to characterize health vulnerability, social vulnerability, and health and social vulnerabilities.<br>Social vulnerability was studied within a 'hazards of place' framework, where social and environmental vulnerabilities interact with risk and responses to generate 'place' vulnerability.<br>"Vulnerability analyses indicate that COVID-19 rates were associated with mental health and chronic conditions along with social determinants that represent inequities in education, income, healthcare access, and race/ethnicity, which may have disproportionately exposed some communities more than others to infection and severe health outcomes."

9	Neto et al. 2022 [59]	Brazil	"…emphasize structural factors associated with the highest COVID-19 vulnerability among sexual and gender minorities, which suggests overlapping vulnerabilities, as described by a syndemic of a mutually caused epidemic."	"Personal, social, and programmatic vulnerabilities quickly worsened in the Brazilian sexual and gender minorities and flagged the pandemic as a syndemic."<br>"Personal vulnerability includes cognitive and behavioural aspects linked to disease awareness and the possibility of change. Social vulnerability refers to social aspects of personal vulnerability (e.g., political decisions and cultural barriers). Programmatic vulnerability constitutes the social level of the government" (e.g., commitment to promote preventive and education actions).<br>"Multiple historical and present-day factors have created the syndemic condition, including lower schooling levels, non-white race/skin colour, worse working conditions or unemployment, and receiving income support during the COVID-19 pandemic."
10	Rogers et al. 2021 [57]	USA	"…syndemics theory can be used as a lens to understand multiple forms of structural and social disadvantage that affect street-based sex workers and their risk of COVID-19 exposure and acquisition."<br>"Multiple and co-occurring risk factors (homelessness, food insecurity, mental health problems, and STIs/HIV) place street-based sex workers at higher risk of COVID-19 and associated social impacts." "The syndemics framework clarifies how multiple factors are associated with increased COVID-19 risk."	"Sex workers represent a high-priority population given their vulnerability and close contact with others, which increases the potential for community spread", but also, they "are considered a vulnerable population for myriad health conditions with notable systemic inequities that perpetuate disadvantage, including COVID-19."<br>"These health disparities are synergistic and best understood through a syndemics lens whereby street-based sex workers are more vulnerable to these conditions as a result of societal marginalization, lack of access to health care, and stigma." "Paradoxically, despite the challenges experienced by this group, they are often forgotten in the identification of vulnerable communities and not considered in relief programs."
11	Siegal et al., 2022 [48]	USA	"COVID-19 interacted with structural racism to further exacerbate existing health disparities in Black communities."<br>"Syndemic theory is well-suited for framing the impacts of COVID-19 and structural racism because it highlights how structural racism interacts with COVID-19 to contribute to racially disparate health outcomes as a result of inequitable access to SDH."<br>"COVID-19 and structural racism exacerbate the negative impacts of the other." "This study used a multicomponent approach, including geospatial analysis, to inform a	"Inequitable access to SDH can affect multiple domains of individuals' and families' lives and, critically, creates differential exposure and vulnerability to health-compromising conditions such as COVID-19."<br>"Structural contexts are used to stratify populations along with their socioeconomic position, based on income, education, race, and ethnicity, which shape individuals' ability to access SDH."<br>"Existing structural inequities create a path through which COVID-19 can disproportionately impact Black families and communities." "The pandemic exacerbated inequities that were already present…"

			contextually adapted, data-driven, cross-sector response to the syndemic.”	
12	Singh et al. 2021 [27]	India	Uses the syndemic framework to understand patients’ experiences of chronic disease care, challenges faced during the lockdown, their coping strategies, and mitigators during the pandemic in the context of socioecological and biological factors.<br>The intersection of biological and socioecological factors contributes to the intensified effects of the pandemic.	“Recognize that socioecological and biological factors contribute to vulnerability to COVID-19 infection among people with chronic conditions.”<br>“Although having a chronic disease increases one’s vulnerability to COVID-19, patients’ worsened health outcomes during the pandemic could be explained by unintended consequences of preventive measures (and lockdowns) to combat the pandemic.”<br>PLWCDs are experiencing a syndemic in which the pandemic exacerbates their pre-existing chronic condition, already occurring alongside other potentially marginalizing sociopolitical and ecological factors, that contribute to poor health outcomes.
13	Wildman et al. 2022 [61]	England	The syndemics’s framework was used “to explore the impacts of the COVID-19 pandemic on the lives of people living with, often multiple, chronic health conditions in a range of social circumstances.”<br>Studied syndemic vulnerability among people living with chronic non-communicable diseases during the COVID-19 pandemic. “Syndemic vulnerability was greatly increased for participants whose chronic ill health placed them at increased risk of harm or death from COVID-19, but who lacked the resources to comfortably shield themselves and their loved ones from harm. The long-term effects of debt and depression are likely to outlast the effects of the pandemic.”	The pandemic public health response increased the work required for condition management in NCD patients.<br>“An acute awareness that their NCDs placed them at increased vulnerability to serious harm from COVID-19 was causing deep anxiety for many participants.” Social isolation was leaving some vulnerable to intrusive thoughts.<br>Practical measures are to be welcomed to allow vulnerable people to stay safe. “As people from ethnic minority backgrounds appear to be at increased vulnerability to COVID-19, their accounts of the syndemic impacts of the pandemic may differ.”
14	Borghi-Silva et al., 2022 [64]	Latin America	“Analysing COVID-19 from the syndemic perspective demonstrates the interaction of multiple comorbidities and the increase of contagion in people who are socially vulnerable.”<br>“…A syndemic perspective can conceptualize a variety of vulnerabilities caused by COVID-19.”	“The number of deaths by COVID-19 in LA is strongly associated with multi-morbidities (diabetes, obesity, sedentary, smoking, among others) and disproportionately attacks communities located in poorer, low-income regions and ethnic minorities.”<br>“The COVID-19 pandemic in Latin America can be considered a syndemic, where factors that affect the health of the population such as comorbidities, economic aspects, housing conditions, nutritional status, lifestyle, and low income all negatively impact health trajectories and increase the spread of COVID-19.”

15	Burigo and Porto, 2021 [32]	Brazil	"The concept of syndemics contextualizes the COVID-19 pandemic in terms of poverty and social injustice, as it also reveals the synergy with other pandemics related to the advancement of the global food system: malnutrition, obesity, and climate change, which all have a strong influence on the dominant model of agriculture."<br>"COVID-19 must be considered a new syndemic, being at the same time a cause and a consequence".<br>The syndemic concept broadens and makes complex those of epidemic and pandemic, while adding the concept of vulnerability.	Vulnerability is a polysemic concept developed by many subjects and fields of knowledge focused on studying themes such as development and sustainability, poverty and food and nutrition security, natural and technological disasters, global climate change, and public health issues, among others. It is applied to analyse why some countries, regions, and population groups have worse consequences or impacts in the face of events with similar characteristics, such as disasters and epidemics.<br>With the outbreak and advancement of the COVID-19 pandemic, pre-existing social inequalities and vulnerabilities have worsened, especially affecting, other than risk groups with comorbidities, certain countries, territories, and populations.
16	Di Ciaula et al., 2021 [33]	Global	"…the pandemic becomes syndemic due to interactions with noncommunicable diseases, climate changes, and inequities."<br>"COVID-19 is not simply a pandemic, it can be considered one of the possible, multiple expressions of a pre-existing syndemic."<br>"Judging COVID-19 as a syndemic requires a syndemic approach not only to provide an appropriate response to the pandemic but also to limit the risk of further pandemics."<br>"...research on the COVID-19 pandemic can benefit from a syndemic approach, which is particularly useful to understand vulnerability and the unequal impacts of this public health crisis."<br>The syndemic aspects emphasize the role of existing inequalities and environmental criticalities.	"Explore the complex relationships existing, mainly in Western countries, between the viral pandemic and factors, which increase vulnerability."<br>"…Obesity, hypertension, cardiovascular diseases, chronic respiratory diseases, and cancer have been spreading across poorer and most vulnerable countries." In Western countries, in particular, there are complex relationships between COVID-19, environmental, and other factors that increase individual vulnerability in subjects at risk.<br>"Underscores the role of NCD as factors increasing individual vulnerability in the pandemic era, and of the pandemic as an amplifier of previously existing risk factors."<br>"The COVID-19 pandemic can hit each area in a different way and with violence, depending on pre-existing factors regulating individual vulnerability and the possibility of recovery."
17	Fronteira et al., 2021 [31]	Global	"Advocate for the adoption of a syndemic policy approach that enables a holistic and distinctive understanding of the intersection of prevalent (endemic) diseases and COVID-19-specific vulnerabilities and disparities experienced by individuals, populations, communities, and societies."<br>"Using as a starting point the syndemic theory that translates the cumulative and intertwined factors between different epidemics, argues that the SARS-CoV-2 is a one health issue of a syndemic nature and that the failure to acknowledge this contributes to weakened policy-making processes and public	"COVID-19-specific factors (e.g., social, biotic, and abiotic environments) and outcomes lead to the clustering of health vulnerabilities and disparities over time."<br>"Further research on syndemics has underscored (i) a need to focus on social inequality as a root cause of syndemic interactions, and (ii) that population-level disease prevention can only occur through addressing the large-scale social forces that shape both individual and population health." "…Emphasize the need for equitable solutions to infectious disease challenges, ensuring that policy response mechanisms and interventions are reflective of the disproportionate disease burdens borne by vulnerable and marginalized populations…"

			health responses and ineffective health policies and programs.”	
18	Garcia, 2022 [51]	USA	Uses syndemics theory to understand the biological-biological interactions of diseases and the biological-social implications for COVID-19 to address health inequities associated with COVID-19 due to systemic racism.<br>“Contextualizing COVID-19 through a syndemic and SDOH framework provides an opportunity to collaborate with public health professionals toward developing biological-social interventions for diverse racial/ethnic minorities and highly marginalized populations.”	“Significant structural barriers (e.g., poverty, unemployment, affordable health care, food and housing insecurity, immigration, and legal status) increase vulnerabilities to health disparities.”<br>“Social stratification contextualizes the pandemic about power and status and highlights the role social structures have in perpetuating health inequities by harnessing resources and protecting the status quo, which increases vulnerabilities for racial/ethnic minorities and highly marginalized populations.”
19	Kalichman and El-Krab 2022 [46]	Global	“The potential for COVID-19’s synergistic relationships with HIV infection and their multiple co-occurring conditions have brought some social and behavioural HIV researchers to conceptualize COVID-19-HIV co-infection within a syndemics framework.”<br>“The COVID-19 pandemic impacts the mental health and structural inequalities of PLWH, and these impacts can be conceptualized within the context of HIV syndemics.”	“PLWHIV are typically attuned to the fact that they can be more vulnerable to an array of infectious diseases.” “They may have been particularly vulnerable to the adverse effects of social isolation given the limited social support they often experience due to HIV stigma and intersecting sources of rejection.”<br>“Synergistic impacts can diminish motivation to engage in COVID-19 mitigation practices, and suppress immune response, increasing vulnerability to severe COVID-19.”
20	Mezzina et al., 2022 [43]	Global	“Covid-19 is a syndemic, in which the consequences of the disease are determined by privilege and a variety of social and material factors, and are exacerbated by social and economic disparity.”	“Vulnerable groups are exposed to a higher risk of illness and have limited personal and social resources to cope with its mental health consequences.” “Social vulnerability refers to the potential negative effects on communities of external stresses on human health, social mobility, and social capital.”<br>“Social factors are inherent to vulnerability, that increase the risk of mental health conditions; in turn, mental ill health can further engender social disadvantage and social exclusion.”
21	Pirrone et al., 2021 [65]	Global	“The focus on the complex interplay among biological and contextual factors characterizes the syndemic approach.” “Syndemic research also calls for multi-level analyses to understand interactions at the individual and population levels.”	“Research on the COVID-19 pandemic can benefit from a syndemics approach, particularly to understand vulnerability and the unequal impacts of this public health crisis.”<br>Contextual factors such as different kinds of inequality, violence, discrimination, exclusion, poverty or economic stress affect

			“NCD-related syndemics research would benefit from further analysis of structural factors and the interconnections between syndemic components across multiple levels, together with more ambitious research designs integrating quantitative and qualitative methods.”	marginalized or vulnerable populations (e.g., people with NCDs and mental health problems).<br>“Incorporating approaches from across disciplines into syndemic research is more crucial than ever because this may help to understand the vulnerability to and the diverse impacts of the COVID-19 pandemic.”
22	Pryor and Dietz, 2022 [35]	USA	The COVID-19, obesity, and the food insecurity syndemic impact conditions required for food sovereignty (e.g., food access, health equity, fair and living wages, land access, just immigration policy, restraints upon corporations, and environmental well-being).	Numerous vulnerabilities and disparities are illustrated by the COVID-19 syndemic along the US food supply chain.<br>“The effects of the COVID-19 pandemic on the food and agriculture system illustrate the vulnerability of the industrialized food system and the disproportionate impact of the pandemic on the workforce essential to the food supply chain.”
23	Singer and Rylco-Bauer 2021 [63]	Global (emphas is in the USA)	“Marrying the concept of ‘synergy’ with ‘epidemic’, the syndemics approach recognizes that diseases in a population occur neither independently of social and ecological conditions nor in isolation from other diseases.”<br>The specific value of the syndemics approach about COVID-19 is that “it focuses attention on three essential intersections in the making of health that have come together to shape this pandemic: 1) synergistic interactions among diseases and other health conditions that increase the overall burden of sickness at different levels beyond that resulting from mere comorbidity; 2) interspecies interactions that lead to emergent and spreading infectious zoonotic diseases; and 3) health and society interactions that support the clustering of multiple diseases and risks in vulnerable populations(...)”	Disenfranchised and vulnerable cultural groups (e.g., black, and Latin population, homeless, immigrants) living in fragile and vulnerable settings (e.g., low-income, limited healthcare and other social infrastructure) have higher rates of infection (more exposed and less protected).<br>Refers to the concept of structural vulnerability and argues that is “produced by [a person’s] location in a hierarchical social order and its diverse networks of power relationships and effects. Individuals are structurally vulnerable when they are subject to structural violence in its broadest conceptualization.”<br>Vulnerability conditions include poor access to quality health care; working in front-line, low wage “essential” jobs; reliance on public transportation, and challenges in following preventive measures. COVID-19 is also impacting different structurally vulnerable groups (e.g., refugees/migrants and women).
24	Ali, 2021 [39]	Pakistan	Syndemic as a perspective to understand interactions among COVID-19, chronic kidney diseases (CKDs), and diabetes.<br>Several specificities of COVID-19 are “due to the entwinement of local historical, sociocultural, environmental, economic, and political factors, and structured vulnerabilities, which caused differences spread and effects of the virus in the country. Medical anthropologists have called this entwinement "syndemics".”	“Evidence makes a low-income country like Pakistan highly vulnerable in the face of a critical outbreak of COVID-19”<br>Institutionalized forms of disparities and NCDs allow COVID-19 to affect countries and add further risks to the already at-risk populations.<br>Factors contributing to structured vulnerabilities could be confounders in establishing interactions between CKD and diabetes.

25	Bispo and Santos, 2021 [66]	Global	COVID-19 is a syndemic, due to the fact that it does not develop in isolation and is not restricted to the biological dimension of the virus's transmissibility.<br>The syndemic framework makes it possible to understand the synergistic interaction between COVID-19, NCD, infectious diseases, and mental health, which results in an increase in incidence and deaths from all causes involved.	Social, political, economic, and environmental factors create and perpetuate vulnerabilities that influence morbidity and mortality in populations.<br>Direct effects resulting from COVID-19 and other related diseases are linked to the worsening of the population's living conditions, affecting with greater intensity the groups in a situation of individual and social vulnerability.
26	Caron and Adegboye, 2021 [50]	Global	"The syndemic approach recognizes how social realities shape individuals' or communities' illness experiences along with the distribution of diseases across populations."<br>"SDoH can interact synergistically and amplify their deleterious effects (e.g., unemployment and homelessness, poor-quality housing and food deserts) thus affecting a population's health status, vulnerability to disease, and health inequities (…)."	Vulnerable and marginalized populations include the elderly, children, disabled, underinsured, socioeconomically disadvantaged, incarcerated, abused and mentally ill, and racial/ethnic minorities.<br>"Focusing political efforts to improve the SDoH may hold the potential to significantly influence the health outcomes for marginalized populations and thereby reduce the synergistic effects among a population's pre-existing health status and SDoH, and create a healthier and more resilient population."
27	Caron and Aytur, 2022 [49]	Global	"The COVID-19 pandemic is a syndemic that is compounded by preexisting underlying disease and the SDOH-exposome".	"…populations residing in environments with structural susceptibilities experience health adversities and syndemics to a greater extent than their less vulnerable counterparts which warrant a review of the contributory upstream determinants". "In addition to a bi-directional relationship between SDoH and a syndemic, there is potential for a bidirectional relationship between the exposome and a syndemic, and a bi-directional relationship between the exposome and SDoH". "Interactions between SDoH and COVID-19 pandemic had different results for marginalized populations and have worsened health outcomes for many in this synergistic pandemic".
28	Chaudhuri, 2022 [53]	Canada	"Characterize COVID-19 as a syndemic as opposed to a pandemic, emphasizing the intersections of the contributing demographic, social, economic, and environmental factors of the pandemic." "Syndemic theory advances the examination of health and healthcare disparities emphasizing the contexts of social and economic systems in these processes." "Syndemics theory provides a critical alternative to conventional systemic reform culture and recognizes how disparities in social realities are accountable for not only shaping the marginal experience but for its distribution across subgroups of populations."	"Even the unique perspectives of reorienting the healthcare culture to its original benevolent foundation appear to sustain in principle the context of ethnic minority populations' vulnerabilities."<br>"Data on ethnic minority groups are routinely aggregated with vulnerabilities and marginalization concerns". However, "in the absence of intentional transformative approaches, cultural variations in normative principles of health equity and digital health would continue to be interpreted as part of generic data, measured by metrics of seemingly homogeneous vulnerabilities."

29	Knipper et al., 2021 [47]	Peru, South Africa, and Syria	Addressing TB, COVID-19, and migration from a syndemic perspective draws systematic attention to comorbidity and the relevance of social and structural context and helps to find solutions.<br>Syndemic theory "highlights the importance of social, political, and structural context as markers of risk, and identifies communities whose bio-social circumstances are contributing to both pandemics."	Particular vulnerabilities are experienced by displaced people and mobile populations, from being overlooked or actively excluded from health and social policies, and from inhabiting environments where some measures are impossible to realize.<br>Local and regional contexts shape the patterns of risk and vulnerability, and the capabilities of health policies and systems to protect and care for the population.
30	Lemke and Brown, 2020 [44]	USA	"Syndemic perspectives can conceptualize the array of endemic and COVID-19-specific vulnerabilities and disparities among Black women and birthing people, and this understanding can lead to high-impact prevention strategies."<br>"Syndemic perspectives can provide researchers with a theoretical grounding to advance scientific inquiry into the interacting, multi-level, and co-occurring maternal health afflictions that had emerged from dynamic macro-level policies and forces that had led to the clustering of health disparities among this population over time."	The COVID-19 pandemic and corresponding policy changes in the U.S. could disproportionately impact vulnerable populations and perpetuate and worsen population-level morbidity and mortality disparities through the novel and exacerbator risks that impact prone populations due to macrolevel racism/discrimination, macroeconomic, healthcare, and other forces. Exacerbating existing socioeconomic disparities among BWBP, increases vulnerabilities, and enhances the disproportional impact of COVID-19 on this population.
31	Lemke et al., 2020 [34]	USA	The unique co-occurrence of health disparities observed among long-haul truck drivers and known COVID-19 infection, morbidity, and mortality risks suggest the possibility of COVID-19-based truck driver syndemic, with widespread implications for public health and safety.	Long-haul truck drivers are especially reliant on and vulnerable to the barriers and resources that characterize their worksite environments. Their individual and occupational characteristics also favour COVID-19 transmission.
32	Meinhart et al., 2021 [42]	Global	"Explores the bi-directional relationships between infectious diseases and GBV to illustrate how their dynamic interplay magnifies the population health burden". "A syndemic lens is critical to developing policies that address both GBV and infectious disease in a manner that sustains progress on gender mainstreaming and transformation."	Clustering of diseases results in adverse interactions that heighten the population's vulnerability and harmful social norms could perpetuate gender inequality and exacerbate GBV in the syndemic response.<br>"Gender-sensitive data collection involves not only sex-disaggregated data but also collecting data on contextual gender norms" to identify individuals who may require additional support.
33	Melamed et al., 2021 [36]	Global	"The syndemic framework explains how the pandemic and its societal effects act particularly to worsen tobacco-related harm in groups with higher smoking rates." "Syndemics is a conceptual approach to understand how pre-pandemic tobacco-related health risks have been interacting with the societal impact of the pandemic and together make the risk of	Structural factors contribute to health disparities among vulnerable groups, which are more likely to experience harm due to the pandemic and increased tobacco use. "Coordinated action is needed to protect vulnerable groups by lowering barriers to tobacco treatment, enforcing smoke-free policies, and integrating tobacco treatment into community, workplace, and healthcare organizations that serve socioeconomically disadvantaged groups.".

			harm from tobacco more pronounced among disadvantaged groups."	
34	Morrissey and Rivera 2021 [54]	USA	"Syndemic theory advances understanding of the interaction and concentration of disease and macro-level sociopolitical and economic forces, including systemic racism and ageism, that have contributed significantly to suffering and mortality during the pandemic and may guide the formulation of public health policy."	"The disproportionate impact of the pandemic on vulnerable older adults suggests that the pandemic has heightened pre-existing precarity and racial inequities across diverse older adult populations."<br>Suggest that "the needs of vulnerable populations are met in a non-discriminatory manner in the implementation of emergency regulations and guidelines."
35	Poteat et al., 2020 [55]	USA	"Racism and its manifestations (e.g., chattel slavery, mortgage redlining, political gerrymandering, lack of Medicaid expansion, employment discrimination, and healthcare provider bias)" created the syndemic condition.<br>Racism "has converged with the COVID-19 pandemic to accelerate exposure, disease, and mortality among black people in America."	"The preponderance of this vulnerable population in occupations, environments, and situations that increase exposure to COVID-19 is grounded in the historical and modern-day structural violence of racism." Racial health inequities are actively produced through anti-black racism institutionalized within the American political system.
36	Shim and Starks, 2021 [45]	USA	The intersection of COVID-19, mental health inequities, and structural racism, and the resulting syndemic, presents numerous policy challenges—and opportunities.	People with serious mental illnesses and substance use disorders are at greater risk of contracting COVID-19 due to the confluence of SDOH and structural vulnerabilities.<br>The vulnerability of essential workers is described in terms of social circumstances, working conditions, and the likelihood of developing psychiatric disorders. The pandemic increased their vulnerability because they continued working.<br>"Addressing these issues in a unified manner, using a syndemic theory approach, can lead to significant progress and effective solutions for otherwise intransigent problems in society."
37	Sönmez et al., 2020 [58]	USA	"Immigrant workers face excess levels of chronic stress and related syndemic risks that are exacerbated by inequities associated with social, political, economic, and environmental conditions."	Factors that add strain to immigrant workers (e.g., heightened uncertainty and insecurity over their future, absence of social support and isolation, and limited or no access to medical care and health insurance), underly chronic health conditions and compromise their immune systems to increase vulnerability to COVID-19 infection, just as harmful coping mechanisms might also do".<br>"Guided by syndemic frameworks, addressing the social and structural factors that have led to the clustering of health and safety risks among immigrant hospitality workers is necessary to alleviate disparities.".

38	Stark et al., 2020 [62]	Global	"GBV and COVID-19 synergistically reinforce the impacts that would otherwise not arise if either epidemic occurred in isolation."<br>A syndemic perspective is applied to examine their shared influence in humanitarian settings because syndemics are also entrenched in the geographical or temporal setting in which they are immersed.	It is important "to identify and support women as a vulnerable group within the syndemic framework of programming and policy."<br>"GBV services are vulnerable to cessation when health service providers attempt to prevent and control infectious diseases without incorporating a gender-sensitive lens."<br>"Given the magnified social and structural violence faced by certain groups of women, programs, and policies should use intersectional praxis."
39	Williams and Vermund, 2021 [54]	USA	Described the "synergistic effect of infectious disease, underlying chronic conditions, and sociopolitical forces including chronic systemic racism."<br>"Syndemic theory provides a useful framework for examining COVID-19 and other diseases that disproportionately affect vulnerable populations."<br>"A syndemic model can be used to examine racial disparities as potentially more applicable to the US."	Vulnerability to COVID-19 in BIPOC' population is compounded by syndemic factors (e.g., disease concentration and interaction; economic disenfranchisement; primary preventative healthcare barriers; and racialized sociopolitical system).<br>A lack of preparedness shows vulnerabilities in healthcare and public health coordination and systems.
40	Yadav et al., 2020 [37]	Global	COVID-19 is a syndemic for people living with NCDs due to a "synergistic pandemic that interacts with various pre-existing medical conditions and social, ecological, and political factors and exacerbates existing NCDs."<br>"The pandemic escalated into a syndemic due to several driving factors".	NCDs and pre-existing factors increase vulnerability to COVID-19, and COVID-19 increases NCD-related risk factors.<br>"Prevailing inequalities in the social determinants of health, including poor social, economic, and environmental conditions have an impact on various aspects of life".

*Reference in original manuscript.

References (in alphabetical order)

1. Ali, I. Syndemics at play: Chronic kidney disease, diabetes and COVID-19 in Pakistan. *Ann. Med.* **2021**, *53*, 581–586. https://doi.org/10.1080/07853890.2021.1910335.
2. Bispo Júnior, J.P.; Dos Santos, D.B. COVID-19 as a syndemic: A theoretical model and foundations for a comprehensive approach in health. *Cad. Saude Publica* **2021**, *37*, e00119021. https://doi.org/10.1590/0102-311X00119021.
3. Borghi-Silva, A.; Back, G.D.; de Araújo, A.S.G.; Oliveira, M.R.; da Luz Goulart, C.; Silva, R.N.; Bassi, D.; Mendes, R.G.; Arena, R. COVID-19 seen from a syndemic perspective: Impact of unhealthy habits and future perspectives to combat these negative interactions in Latin America. *Prog. Cardiovasc. Dis.* **2022**, *71*, 72–78. https://doi.org/10.1016/j.pcad.2022.04.006.

4. Burigo, A.C.; Porto, M.F. 2030 agenda, health and food systems in times of syndemics: From vulnerabilities to necessary changes. *Cienc. Saude Coletiva* **2021**, *26*, 4411–4424. https://doi.org/10.1590/1413-812320212610.13482021.
5. Caron, R.M.; Adegboye, A.R.A. COVID-19: A Syndemic Requiring an Integrated Approach for Marginalized Populations. *Front. Public Health* **2021**, *9*, 675280. https://doi.org/10.3389/fpubh.2021.675280.
6. Caron, R.M.; Aytur, S.A. Assuring Healthy Populations During the COVID-19 Pandemic: Recognizing Women's Contributions in Addressing Syndemic Interactions. *Front. Public Health* **2022**, *10*, 856932. https://doi.org/10.3389/fpubh.2022.856932.
7. Chaudhuri, E.R. The Syndemic of Inequity and COVID-19 in Virtual Care. *J. Med. Internet Res.* **2022**, *24*, e37717. https://doi.org/10.2196/37717.
8. Chung, G.K.K.; Chan, S.M.; Chan, Y.H.; Yip, T.C.F.; Ma, H.M.; Wong, G.L.H.; Chung, R.Y.N.; Wong, H.; Wong, S.Y.S.; Yeoh, E.K.; et al. Differential impacts of multimorbidity on COVID-19 severity across the socioeconomic ladder in Hong Kong: A syndemic perspective. *Int. J. Environ. Res. Public Health* **2021**, *18*, 8168. https://doi.org/10.3390/ijerph18158168.
9. Cokley, K.; Krueger, N.; Cunningham, S.R.; Burlew, K.; Hall, S.; Harris, K.; Castelin, S.; Coleman, C. The COVID-19/racial injustice syndemic and mental health among Black Americans: The roles of general and race-related COVID worry, cultural mistrust, and perceived discrimination. *J. Community Psychol.* **2022**, *50*, 2542–2561. https://doi.org/10.1002/jcop.22747.
10. Daboin, B.E.G.; Bezerra, I.M.P.; Morais, T.C.; Portugal, I.; Echeimberg, J.D.O.; Cesar, A.E.M.; Cavalcanti, M.P.E.; Jacintho, L.C.; Raimundo, R.D.; Elmusharaf, K.; et al. Deciphering Multifactorial Correlations of COVID-19 Incidence and Mortality in the Brazilian Amazon Basin. *Int. J. Environ. Res. Public Health* **2022**, *19*, 1153. https://doi.org/10.3390/ijerph19031153.
11. Di Ciaula, A.; Krawczyk, M.; Filipiak, K.J.; Geier, A.; Bonfrate, L.; Portincasa, P. Noncommunicable diseases, climate change and iniquities: What COVID-19 has taught us about syndemic. *Eur. J. Clin. Investig.* **2021**, *51*, 13682. https://doi.org/10.1111/eci.13682.
12. Duby, Z.; Bunce, B.; Fowler, C.; Bergh, K.; Jonas, K.; Dietrich, J.J.; Govindasamy, D.; Kuo, C.; Mathews, C. Intersections between COVID-19 and socio-economic mental health stressors in the lives of South African adolescent girls and young women. *Child Adolesc. Psychiatry Ment. Health* **2022**, *16*, 23. https://doi.org/10.1186/s13034-022-00457-y.
13. Fronteira, I.; Sidat, M.; Magalhães, J.P.; de Barros, F.P.C.; Delgado, A.P.; Correia, T.; Daniel-Ribeiro, C.T.; Ferrinho, P. The SARS-CoV-2 pandemic: A syndemic perspective. *One Health* **2021**, *12*, 100228. https://doi.org/10.1016/j.onehlt.2021.100228.
14. Garcia, M. This Is America: Systemic Racism and Health Inequities Amidst the COVID-19 Pandemic. *Soc. Work Public Health* **2022**, *37*, 105–121. https://doi.org/10.1080/19371918.2021.1981509.
15. Islam, N.; Lacey, B.; Shabnam, S.; Erzurumluoglu, A.M.; Dambha-Miller, H.; Chowell, G.; Kawachi, I.; Marmot, M. Social inequality the syndemic of chronic disease COVID-19: County-level analysis in the USA. *J. Epidemiol. Community Health (1978)* **2021**, *75*, 496–500. https://doi.org/10.1136/jech-2020-215626.
16. Jenkins, E.K.; McAuliffe, C.; Hirani, S.; Richardson, C.; Thomson, K.C.; McGuinness, L.; Morris, J.; Kousoulis, A.; Gadermann, A. A portrait of the early and differential mental health impacts of the COVID-19 pandemic in Canada: Findings from the first wave of a nationally representative cross-sectional survey. *Prev. Med.* **2021**, *145*, 106333. https://doi.org/10.1016/j.ypmed.2020.106333.
17. Jenkins, E.K.; Slemon, A.; Richardson, C.; Pumarino, J.; McAuliffe, C.; Thomson, K.C.; Goodyear, T.; Daly, Z.; McGuinness, L.; Gadermann, A. Mental Health Inequities Amid the COVID-19 Pandemic: Findings From Three Rounds of a Cross-Sectional Monitoring Survey of Canadian Adults. *Int. J. Public Health* **2022**, *67*, 1604685. https://doi.org/10.3389/ijph.2022.1604685.
18. Kalichman, S.C.; El-Krab, R. Social and Behavioral Impacts of COVID-19 on People Living with HIV: Review of the First Year of Research. *Curr. HIV/AIDS Rep.* **2022**, *19*, 54–75. https://doi.org/10.1007/s11904-021-00593-8.
19. Knipper, M.; Sedas, A.C.; Keshavjee, S.; Abbara, A.; Almhawish, N.; Alashawi, H.; Lecca, L.; Wilson, M.; Zumla, A.; Abubakar, I.; et al. The need for protecting and enhancing TB health policies and services for forcibly displaced and migrant populations during the ongoing COVID-19 pandemic. *Int. J. Infect. Dis.* **2021**, *113*, S22–S27. https://doi.org/10.1016/j.ijid.2021.03.047.

20. Lee, J.; Ramírez, I.J. Geography of Disparity: Connecting COVID-19 Vulnerability and Social Determinants of Health in Colorado. *Behav. Med.* **2022**, *48*, 72–84. https://doi.org/10.1080/08964289.2021.2021382.
21. Lemke, M.K.; Apostolopoulos, Y.; Sönmez, S. A novel COVID-19 based truck driver syndemic? Implications for public health, safety, and vital supply chains. *Am. J. Ind. Med.* **2020**, *63*, 659–662. https://doi.org/10.1002/ajim.23138.
22. Lemke, M.K.; Brown, K.K. Syndemic Perspectives to Guide Black Maternal Health Research and Prevention During the COVID-19 Pandemic. *Matern. Child Health J.* **2020**, *24*, 1093–1098. https://doi.org/10.1007/s10995-020-02983-7.
23. Meinhart, M.; Vahedi, L.; Carter, S.E.; Poulton, C.; Mwanze Palaku, P.; Stark, L. Gender-based violence and infectious disease in humanitarian settings: Lessons learned from Ebola, Zika, and COVID-19 to inform syndemic policy making. *Confl. Health* **2021**, *15*, 84. https://doi.org/10.1186/s13031-021-00419-9.
24. Melamed, O.C.; Zawertailo, L.; Schwartz, R.; Buckley, L.; Selby, P. Protection des groupes vulnérables contre les méfaits du tabac pendant et après la pandémie de COVID-19. *Health Promot. Chronic Dis. Prev. Can.* **2021**, *41*, 282–287. https://doi.org/10.24095/hpcdp.41.10.02.
25. Mezzina, R.; Gopikumar, V.; Jenkins, J.; Saraceno, B.; Sashidharan, S.P. Social Vulnerability and Mental Health Inequalities in the "Syndemic": Call for Action. *Front. Psychiatry* **2022**, *13*, 894370. https://doi.org/10.3389/fpsyt.2022.894370.
26. Morrissey, M.B.Q.; Rivera, J.L. Protecting the Public's Health in Pandemics: Reflections on Policy Deliberation and the Role of Civil Society in Democracy. *Front. Public Health* **2021**, *9*, 678210. https://doi.org/10.3389/fpubh.2021.678210.
27. Neto, A.O.M.E.; Da Silva, S.A.G.; Gonçalves, G.P.; Torres, J.L. COVID-19 vulnerability among Brazilian sexual and gender minorities: A cross-sectional study. *Cad. Saude Publica* **2022**, *38*, e00234421. https://doi.org/10.1590/0102-311XEN234421.
28. Pirrone, I.; Dieleman, M.; Reis, R.; Pell, C. Syndemic contexts: Findings from a review of research on non-communicable diseases and interviews with experts. *Glob. Health Action* 2021, 14. https://doi.org/10.1080/16549716.2021.1927332.
29. Poteat, T.; Millett, G.A.; Nelson, L.R.E.; Beyrer, C. Understanding COVID-19 risks and vulnerabilities among black communities in America: The lethal force of syndemics. *Ann. Epidemiol.* **2020**, *47*, 1–3. https://doi.org/10.1016/j.annepidem.2020.05.004.
30. Pryor, S.; Dietz, W. The COVID-19, Obesity, and Food Insecurity Syndemic. *Curr. Obes. Rep.* **2022**, *11*, 70–79. https://doi.org/10.1007/s13679-021-00462-w.
31. Rogers, B.G.; Paradis-Burnett, A.; Nagel, K.; Yolken, A.; Strong, S.H.; Arnold, T.; Napoleon, S.C.; Maynard, M.; Sosnowy, C.; Murphy, M.; et al. Sex Workers and Syndemics: A Population Vulnerable to HIV and COVID-19. *Arch. Sex. Behav.* **2021**, *50*, 2007–2016. https://doi.org/10.1007/s10508-021-01940-x.
32. Shim, R.S.; Starks, S.M. COVID-19, structural racism, and mental health inequities: Policy implications for an emerging syndemic. *Psychiatr. Serv.* **2021**, *72*, 1193–1198. https://doi.org/10.1176/appi.ps.202000725.
33. Siegal, R.; Cooper, H.; Capers, T.; Kilmer, R.P.; Cook, J.R.; Garo, L. Using geographic information systems to inform the public health response to COVID-19 and structural racism: The role of place-based initiatives. *J. Community Psychol.* **2022**, *50*, 2611–2629. https://doi.org/10.1002/jcop.22771.
34. Singer, M.; Rylko-Bauer, B. The Syndemics and Structural Violence of the COVID Pandemic: Anthropological Insights on a Crisis. *Open Anthropol. Res.* **2021**, *1*, 7–32. https://doi.org/10.1515/opan-2020-0100.
35. Singh, K.; Kaushik, A.; Johnson, L.; Jaganathan, S.; Jarhyan, P.; Deepa, M.; Kong, S.; Venkateshmurthy, N.S.; Kondal, D.; Mohan, S.; et al. Patient experiences and perceptions of chronic disease care during the COVID-19 pandemic in India: A qualitative study. *BMJ Open* **2021**, *11*, e048926. https://doi.org/10.1136/BMJOPEN-2021-048926.
36. Sönmez, S.; Apostolopoulos, Y.; Lemke, M.K.; Hsieh, Y.C.J. Understanding the effects of COVID-19 on the health and safety of immigrant hospitality workers in the United States. *Tour. Manag. Perspect.* **2020**, *35*, 100717. https://doi.org/10.1016/j.tmp.2020.100717.
37. Stark, L.; Meinhart, M.; Vahedi, L.; Carter, S.E.; Roesch, E.; Scott Moncrieff, I.; Palaku, P.M.; Rossi, F.; Poulton, C. The syndemic of COVID-19 and gender-based violence in humanitarian settings: Leveraging lessons from Ebola in the Democratic Republic of Congo. *BMJ Glob. Health* **2020**, *5*, e004194. https://doi.org/10.1136/bmjgh-2020-004194.

38. Wildman, J.M.; Morris, S.; Pollard, T.; Gibson, K.; Moffatt, S. "I wouldn't survive it, as simple as that": Syndemic vulnerability among people living with chronic non-communicable disease during the COVID-19 pandemic. *SSM Qual. Res. Health* **2022**, *2*, 100032. https://doi.org/10.1016/j.ssmqr.2021.100032.
39. Williams, C.; Vermund, S.H. Syndemic Framework Evaluation of Severe COVID-19 Outcomes in the United States: Factors Associated with Race and Ethnicity. *Front. Public Health* **2021**, *9*, 720264. https://doi.org/10.3389/fpubh.2021.720264.
40. Yadav, U.N.; Rayamajhee, B.; Mistry, S.K.; Parsekar, S.S.; Mishra, S.K. A Syndemic Perspective on the Management of Non-communicable Diseases Amid the COVID-19 Pandemic in Low- and Middle-Income Countries. *Front. Public Health* **2020**, *8*, 00508. https://doi.org/10.3389/fpubh.2020.00508.
